# Supplementary material for: An Integrated Perspective on Spatio-Temporal Attention and Infant Language Acquisition
Source: Int J Environ Res Public Health. 2021 Feb 8;18(4):1592. doi: 10.3390/ijerph18041592 (PMC7915013; doi:10.3390/ijerph18041592)
Supplement: Supplementary file 1 [file ijerph-18-01592-s001.pdf]

Supplementary materials

The sample analyzed in experiment 1 is part of a larger cohort of infants participating in our study. The original sample included 48 infants (23 F, mean age in days = 120, SD = 14). Twenty-four infants were excluded because they show less than three valid trials per condition. Figure S1 shows the descriptive statistics of saccade latency (in ms) for the remaining 24 subjects: a) by condition, b) by position of S2 (i.e., left and right of S1) and c) by subject. The results overlap those presented in experiment 1.

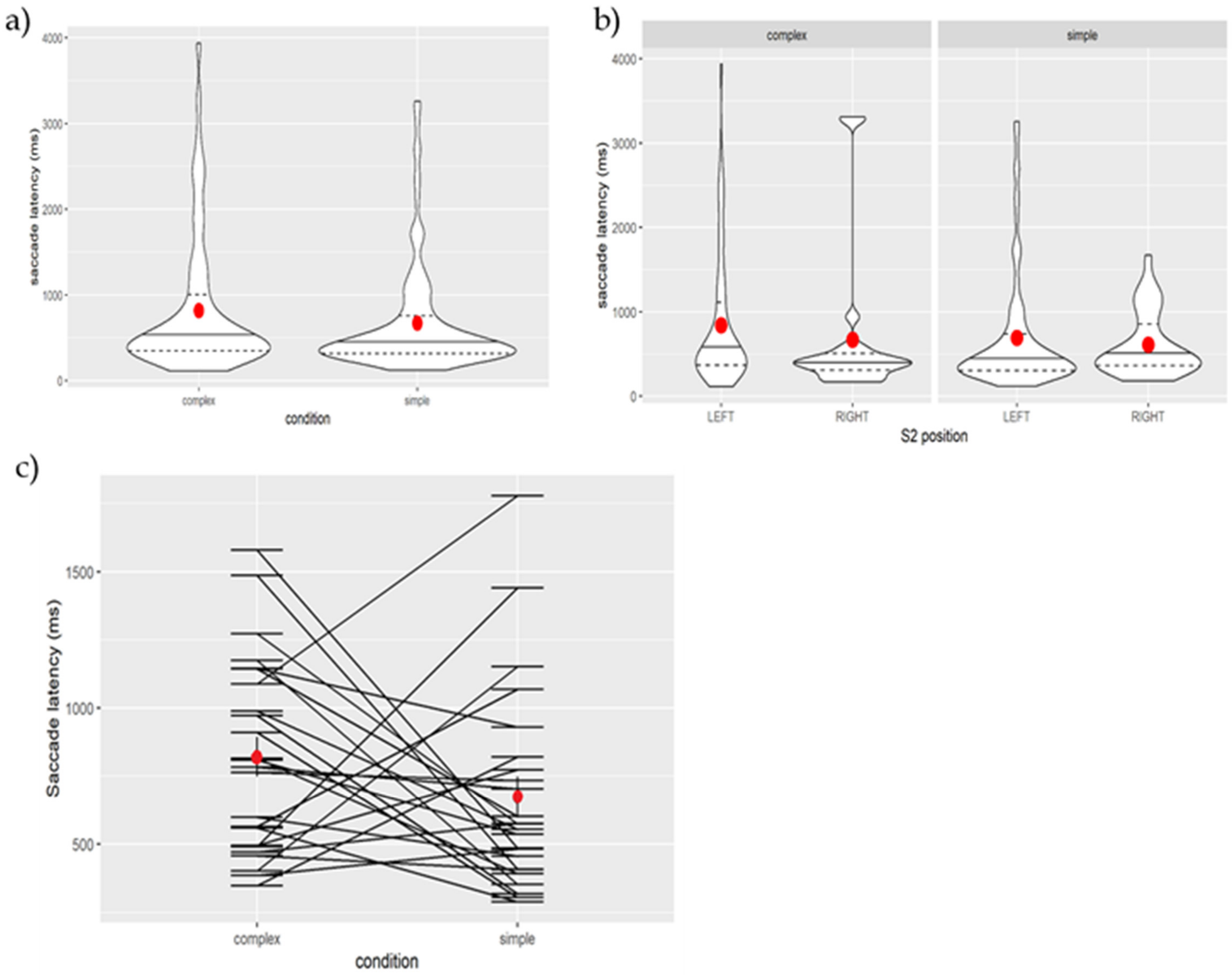

Figure S1. Descriptive statistics of saccade latency (in ms) for the complete sample of 24 subjects participating in the experiment 1.
